# A characterization of seminormal C-monoids

**DOI:** 10.1007/s40574-019-00194-9

**Published:** 2019-02-09

**Authors:** Alfred Geroldinger, Qinghai Zhong

**Affiliations:** Institute for Mathematics and Scientific Computing, NAWI Graz, University of Graz, Heinrichstraße 36, 8010 Graz, Austria

**Keywords:** Krull monoids, C-monoids, Seminormal, Class semigroups, Half-factorial, 20M13, 13A05, 13A15, 13F05, 13F45

## Abstract

It is well-known that a C-monoid is completely integrally closed if and only if its reduced class semigroup is a group and if this holds, then the C-monoid is a Krull monoid and the reduced class semigroup coincides with the usual class group of Krull monoids. We prove that a C-monoid is seminormal if and only if its reduced class semigroup is a union of groups. Based on this characterization we establish a criterion (in terms of the class semigroup) when seminormal C-monoids are half-factorial.

## Introduction

A C-monoid *H* is a submonoid of a factorial monoid, say $H \subset F$, such that $H^{\times } = H \cap F^{\times }$ and the reduced class semigroup is finite. A commutative ring is a C-ring if its multiplicative monoid of regular elements is a C-monoid. Every C-monoid is Mori (i.e., *v*-noetherian), its complete integral closure ${\widehat{H}}$ is a Krull monoid with finite class group ${\mathcal {C}} ({\widehat{H}})$, and the conductor $(H \negthinspace : \negthinspace {\widehat{H}})$ is non-trivial. Conversely, every Mori domain *R* with non-zero conductor ${\mathfrak {f}} = (R \negthinspace : \negthinspace {\widehat{R}})$, for which the residue class ring ${\widehat{R}}/{\mathfrak {f}}$ and the class group ${\mathcal {C}} ({\widehat{R}})$ are finite, is a C-domain ([10, Theorem 2.11.9]), and these two finiteness conditions are equivalent to being a C-domain for non-local semilocal noetherian domains ([23, Corollary 4.5]). The following result is well-known (see Sect. 2 where we gather the basics on C-monoids).

### Theorem A

Let *H* be a C-monoid. Then *H* is completely integrally closed if and only if its reduced class semigroup is a group. If this holds, then *H* is a Krull monoid and the reduced class semigroup coincides with the class group of *H*.

The goal of the present note is the following characterization of seminormal C-monoids. Note that all seminormal C-domains are seminormal Mori domains (see [2] for a survey) and that, in particular, all seminormal orders in algebraic number fields (see [7]) are seminormal C-domains. Moreover, the seminormalization of any C-monoid is a seminormal C-monoid (Proposition 3.1).

### Theorem 1.1

Let *H* be a C-monoid. Then *H* is seminormal if and only if its reduced class semigroup is a union of groups. If this holds, then we have

1. The class of every element of *H* is an idempotent of the class semigroup.
2. The constituent group of the smallest idempotent of the class semigroup (with respect to the Rees order) is isomorphic to the class group of the complete integral closure of *H*.

The finiteness of the class semigroup allows to establish a variety of arithmetical finiteness results for C-monoids (for a sample, out of many, see [10, Theorem 4.6.6] or [8]). In the special case of Krull monoids with finite class group, precise arithmetical results can be given in terms of the structure of the class group (for a survey see [25]). As a first step in this direction in the more general case of seminormal C-monoids, we establish—based on Theorem 1.1—a characterization of half-factoriality in terms of the class semigroup (Theorem 4.2).

## Background on C-monoids

We denote by ${\mathbb {N}}$ the set of positive integers and set ${\mathbb {N}}_0 = {\mathbb {N}}\cup \{0\}$. For rationals $a, b \in {\mathbb {Q}}$, we denote by $[a,b] = \{x \in {\mathbb {Z}}\mid a \le x \le b\}$ the discrete interval lying between *a* and *b*. All semigroups and rings in this paper are commutative and have an identity element and all homomorphisms respect identity elements. Let *S* be a multiplicatively written semigroup. Then $S^{\times }$ denotes its group of invertible elements and ${\mathsf {E}} (S)$ its set of idempotents. For subsets $A, B \subset S$ and an element $c \in S$ we set

$$\begin{aligned} AB = \{ab \mid a \in A, b \in B \} \quad \text {and} \quad cB = \{cb \mid b \in B \} \,. \end{aligned}$$

Let ${\mathcal {C}}$ be a semigroup. We use additive notation and have class groups and class semigroups in mind. Let $e, f \in {\mathsf {E}} ({\mathcal {C}})$. We denote by ${\mathcal {C}}_e$ the set of all $x \in {\mathcal {C}}$ such that $x+e=x$ and $x+y=e$ for some $y \in {\mathcal {C}}$. Then ${\mathcal {C}}_e$ is a group with identity element *e*, called the *constituent group* of *e*. If $e \ne f$, then ${\mathcal {C}}_e \cap {\mathcal {C}}_f = \emptyset$. An element $x \in {\mathcal {C}}$ is contained in a subgroup of ${\mathcal {C}}$ if and only if there is some $e \in {\mathsf {E}} ({\mathcal {C}})$ such that $x \in {\mathcal {C}}_e$. Thus ${\mathcal {C}}$ is a union of groups if and only if

2.1 $$\begin{aligned} {\mathcal {C}} = \bigcup _{e \in {\mathsf {E}} ({\mathcal {C}})} {\mathcal {C}}_e \,. \end{aligned}$$

Semigroups with this property are called *Clifford semigroups* ([15, 16]). Clearly, $0 \in {\mathsf {E}} ({\mathcal {C}})$ and ${\mathcal {C}}_0 = {\mathcal {C}}^{\times }$. The Rees order $\le$ on ${\mathsf {E}} (S)$ is defined by $e \le f$ if $e+f = e$. If ${\mathsf {E}} (S) = \{0=e_0, \ldots , e_n \}$, then $e_0$ is the largest element and $e_0 + \cdots + e_n$ is the smallest idempotent in the Rees order. If ${\mathcal {C}}$ is finite, then for every $a \in {\mathcal {C}}$ there is an $n \in {\mathbb {N}}$ such that $na = a + \cdots + a \in {\mathsf {E}} ({\mathcal {C}})$.

By a *monoid*, we mean a cancellative semigroup. We use multiplicative notation and have monoids of nonzero elements of domains in mind. Let *H* be a monoid, $H_{{\text {red}}} = \{aH^{\times } \mid a \in H\}$ the associated reduced monoid, and ${\mathsf {q}} (H)$ the quotient group of *H*. We denote by

- $H' = \{ x \in {\mathsf {q}} (H) \mid$ there is an $m \in {\mathbb {N}}$ such that $x^n \in H$ for all $\ n \ge m \}$ the *seminormalization*,
- ${\widetilde{H}} = \{ x \in {\mathsf {q}} (H) \mid$ there is an $N \in {\mathbb {N}}$ such that $\ x^N \in H \}$ the *root closure*, and by
- ${\widehat{H}} = \{ x \in {\mathsf {q}} (H) \mid$ there is a $c \in H$ such that $cx^n \in H$ for all $\ n \in {\mathbb {N}}\}$ the *complete integral closure* of *H*.

Then $H \subset H' \subset {\widetilde{H}} \subset {\widehat{H}} \subset {\mathsf {q}} (H)$ and *H* is said to be

- *seminormal* if $$H = H'$$,
- *root closed* if $H = {\widetilde{H}}$,
- *completely integrally closed* if $H = {\widehat{H}}$,
- *v*-*noetherian* (or *Mori*) if H satisfies the ACC on divisorial ideals,
- a *Krull monoid* if H is a completely integrally closed Mori monoid.

Let *F* be a factorial monoid, say $F = F^{\times } \times {\mathcal {F}} (P)$, where ${\mathcal {F}} (P)$ is the free abelian monoid with basis *P*. Then every $a \in F$ can be written in the form $a = \varepsilon \prod _{p \in P} p^{{\mathsf {v}}_p (a)}$, where $\varepsilon \in F^{\times }$ and ${\mathsf {v}}_p :F \rightarrow {\mathbb {N}}_0$ is the *p*-adic exponent. Let $H \subset F$ be a submonoid. Two elements $y, y' \in F$ are called *H*-equivalent (we write $y \sim y'$) if $y^{-1}H \cap F = {y'}^{-1} H \cap F$ or, in other words,

$$\begin{aligned} \text {if for all} \ x \in F, \ \text {we have} \ xy \in H \quad \text {if and only if} \quad xy' \in H \,. \end{aligned}$$

*H*-equivalence defines a congruence relation on *F* and for $y \in F$, let $[y]_H^F = [y]$ denote the congruence class of *y*. Then

$$\begin{aligned} {\mathcal {C}} (H,F) = \{[y] \mid y \in F \} \quad \text {and} \quad {\mathcal {C}} ^* (H,F) = \{ [y] \mid y \in (F {\setminus } F^{\times }) \cup \{1\} \} \end{aligned}$$

are commutative semigroups with identity element [1] (introduced in [9, Section 4]). ${\mathcal {C}} (H,F)$ is the *class semigroup* of *H* in *F* and the subsemigroup ${\mathcal {C}}^* (H,F) \subset {\mathcal {C}} (H,F)$ is the *reduced class semigroup* of *H* in *F*. We have

2.2 $$\begin{aligned} {\mathcal {C}} (H,F) = {\mathcal {C}}^* (H,F) \cup \{[y] \mid y \in F^{\times }\} \quad \ \text {and}\quad \ \{[y] \mid y \in F^{\times }\} \cong F^{\times }/H^{\times } \,. \end{aligned}$$

It is easy to check that either $\{[y] \mid y \in F^{\times } \} \subset {\mathcal {C}}^* (H,F)$ or ${\mathcal {C}}^* (H,F) \cap \{[y] \mid y \in F^{\times } \} = \{[1]\}$. Thus

2.3 $$\begin{aligned} {\mathcal {C}} (H,F) \text { is a union of groups if and only if } {\mathcal {C}}^* (H,F) \text { is a union of groups} \end{aligned}$$

and ${\mathcal {C}} (H,F)$ is finite if and only if both ${\mathcal {C}}^* (H,F)$ and $F^{\times }/H^{\times }$ are finite. As usual, (reduced) class semigroups and class groups will be written additively.

A monoid homomorphism $\partial :H \rightarrow {\mathcal {F}} (P)$ is called a *divisor theory* (for *H*) if the following two conditions hold:

- If $a, b \in H$ and $\varphi (a) \mid \varphi (b)$ in ${\mathcal {F}} (P)$, then $a \mid b$ in *H*.
- For every $\alpha \in {\mathcal {F}} (P)$ there are $a_1, \ldots , a_m \in H$ such that $\alpha = \gcd \big (\varphi (a_1), \ldots , \varphi (a_m) \big )$.

A monoid has a divisor theory if and only if it is a Krull monoid ( [10, Theorem 2.4.8]). Suppose *H* is a Krull monoid. Then there is an embedding of $H_{{\text {red}}}$ into a free abelian monoid, say $H_{{\text {red}}} \hookrightarrow {\mathcal {F}} (P)$. In this case ${\mathcal {F}} (P)$ is uniquely determined (up to isomorphism) and

$$\begin{aligned} {\mathcal {C}} (H) = {\mathsf {q}} ({\mathcal {F}} (P))/ {\mathsf {q}} (H_{{\text {red}}}) \end{aligned}$$

is the (divisor) class group of *H* which is isomorphic to the *v*-class group ${\mathcal {C}}_v (H)$ of *H* ([10, Sections 2.1 and 2.4]).

A monoid *H* is a C-*monoid* if it is a submonoid of some factorial monoid $F = F^{\times } \times {\mathcal {F}} (P)$ such that $H^{\times } = H \cap F^{\times }$ and the reduced class semigroup is finite. We say that *H* is *dense* in *F* if ${\mathsf {v}}_p (H) \subset {\mathbb {N}}_0$ is a numerical monoid for all primes *p* of *F*. Suppose that *H* is a C-monoid. Proofs of the following facts can be found in ([10, Theorems 2.9.11 and 2.9.12]). The monoid *H* is *v*-noetherian, the conductor $(H \negthinspace : \negthinspace {\widehat{H}}) \ne \emptyset$, and there is a factorial monoid *F* such that $H \subset F$ is dense. Suppose that $H \subset F$ is dense. Then the map

2.4 $$\begin{aligned} \partial :{\widehat{H}} \rightarrow {\mathcal {F}} (P), \quad \text {defined by} \quad \partial (a) = \prod _{p \in P} p^{{\mathsf {v}}_p (a)} \end{aligned}$$

is a divisor theory. In particular, ${\widehat{H}}$ is a Krull monoid, $F_{{\text {red}}}$ and hence ${\mathcal {C}}^* (H,F)$ are uniquely determined by *H*, and we call ${\mathcal {C}}^* (H,F)$*the reduced class semigroup of H*.

2.5 $$\begin{aligned} \text {If }{\mathcal {C}}^* (H,F) \text { is a group, then }H \text { is a Krull monoid} \end{aligned}$$

and every Krull monoid with finite class group is a C-monoid.

Let *R* be a (commutative integral) domain. Then *R* is seminormal (completely integrally closed, Mori) if and only if its multiplicative monoid $R^{\bullet }$ of nonzero elements has the respective property. Moreover, *R*

- is a *Krull domain* if $R^{\bullet }$ is a Krull monoid, and
- is a *C-domain* if $R^{\bullet }$ is a C-monoid.

Both statements generalize to rings with zero-divisors ( [12, Theorem 3.5 and Section 4]). We refer to [1, 6, 17, 20] for C-monoids, that do not stem from ring theory, and to [18] for a more general concept.

## A characterization of seminormality

In this section we first show that the seminormalization and the root closure of C-monoids are still C-monoids. Since the seminormalization of a monoid is seminormal and the root closure is root closed and hence seminormal, Proposition 3.1 shows that the natural closure operations, seminormalization and root closure, of C-monoids lead to seminormal C-monoids. Then we prove the characterization of seminormality, as formulated in Theorem 1.1. After that we discuss a special class of C-monoids, namely the monoid of product-one sequences over a finite group.

### Proposition 3.1

Let $H \subset F = F^{\times } \times {\mathcal {F}} (P)$ be a C-monoid. Then the canonical maps

$$\begin{aligned} \psi ' :{\mathcal {C}}^* (H,F) \rightarrow {\mathcal {C}}^* (H', F) \quad \text {and} \quad {\widetilde{\psi }} :{\mathcal {C}}^* (H,F) \rightarrow {\mathcal {C}}^* ({\widetilde{H}}, F) \,, \end{aligned}$$

mapping $[a]_H^F \mapsto [a]_{H'}^F$ and $[a]_H^F \mapsto [a]_{{\widetilde{H}}}^F$ for every $a \in F$, are epimorphism with $\psi ' \big ({\mathcal {C}}^* (H,F) \big ) = {\mathcal {C}}^* (H', F)$ and ${\widetilde{\psi }} \big ({\mathcal {C}}^* (H,F) \big ) = {\mathcal {C}}^* ({\widetilde{H}}, F)$. Moreover, $H' \subset F$ is a seminormal C-monoid and ${\widetilde{H}} \subset F$ is a root closed C-monoid. If every class of ${\mathcal {C}}^* (H, F)$ contains an element from *P*, then every class of ${\mathcal {C}}^* (H', F)$ and every class of ${\mathcal {C}}^* ({\widetilde{H}}, F)$ contains an element from *P*.

### Proof

We have $H\subset H'\subset \widetilde{H}\subset F$, $H'$ is seminormal, ${\widetilde{H}}$ is root closed, $H^{\times } = H \cap F^{\times }$, $(H')^{\times }\subset H'\cap F^{\times }$, and $(\widetilde{H})^{\times }\subset \widetilde{H}\cap F^{\times }$. Let $a\in H'\cap F^{\times }$. Then $a^k\in H\cap F^{\times }=H^{\times }$ for some $k\in {\mathbb {N}}$ and hence there exists $b\in H^{\times }\subset H'$ such that $a^kb=1$. Since $a^{k-1}b\in H'$, we obtain that $a\in (H')^{\times }$. Therefore $(H')^{\times }= H'\cap F^{\times }$. Let $a\in \widetilde{H}\cap F^{\times }$. Then $a^k\in H\cap F^{\times }=H^{\times }$ for some $k\in {\mathbb {N}}$ and hence there exists $b\in H^{\times }\subset \widetilde{H}$ such that $a^kb=1$. Since $a^{k-1}b\in \widetilde{H}$, we obtain that $a\in (\widetilde{H})^{\times }$. Therefore $(\widetilde{H})^{\times }=\widetilde{H}\cap F^{\times }$.

To verify that $\psi '$ is well-defined, let $a, b \in F$ with $[a]_H^F=[b]_H^F$. For an $x\in F$ with $ax\in H'$ we have to verify that $bx\in H'$. Then this implies that $[a]_{H'}^F=[b]_{H'}^F$. In fact, since $ax\in H'$, there exists $N\in {\mathbb {N}}$ such that for all $n\ge N$, we have $(ax)^n\in H$. Since $[a]_H^F=[b]_H^F$, it follows that $[a^n]_H^F=[b^n]_H^F$ whence $a^nx^n \in H$ implies that $b^nx^n\in H$. Therefore for all $n\ge N$, we have $(bx)^n\in H$ which implies that $bx\in H'$.

To verify that $\widetilde{\psi }$ is well-defined, let $a, b \in F$ with $[a]_H^F=[b]_H^F$ and let $x\in F$ such that $ax\in \widetilde{H}$. Since $ax\in \widetilde{H}$, there exists $N\in {\mathbb {N}}$ such that $(ax)^N\in H$. Since $[a]_H^F=[b]_H^F$, it follows that $[a^N]_H^F=[b^N]_H^F$ whence $a^Nx^N \in H$ implies that $b^Nx^N\in H$. Therefore $bx\in \widetilde{H}$.

Obviously, $\psi '$ and $\widetilde{\psi }$ are epimorphisms with

$$\begin{aligned} \begin{aligned} \psi ' ({\mathcal {C}}^*(H,F))&=\psi ' (\{[a]_H^F\mid a\in F{\setminus } F^{\times }\cup \{1\}\})=\{[a]_{H'}^F\mid a\in F{\setminus } F^{\times } \cup \{1\} \} = {\mathcal {C}}^* (H',F) \quad \text {and} \\ \widetilde{\psi } ({\mathcal {C}}^*(H,F))&= \widetilde{\psi } (\{[a]_H^F\mid a\in F{\setminus } F^{\times }\cup \{1\}\})=\{[a]_{\widetilde{H}}^F\mid a\in F{\setminus } F^{\times } \cup \{1\} \}={\mathcal {C}}^*(\widetilde{H},F) \,. \end{aligned} \end{aligned}$$

Thus $H' \subset F$ is a seminormal C-monoid and ${\widetilde{H}} \subset F$ is a root closed C-monoid. $\square$

### Proof of Theorem 1.1

By [10, Theorem 2.9.11.4], we may choose a special factorial monoid *F* such that $H \subset F$ is a dense C-monoid. This special choice will turn out to be useful in the proof of part 3. Since ${\widehat{H}}$ is a Krull monoid, we have ${\widehat{H}} = {\widehat{H}}^{\times } \times H_0$, where $H_0$ is a reduced Krull monoid. If the embedding $H_0 \hookrightarrow {\mathcal {F}} (P)$ is a divisor theory, then $H \subset F = {\widehat{H}}^{\times } \times {\mathcal {F}} (P)$ is a dense C-monoid. In particular, we have $H^{\times } = H \cap F^{\times }$ and ${\mathcal {C}}^* = {\mathcal {C}}^* (H,F)$ is finite. Furthermore, there are $\alpha \in {\mathbb {N}}$ and a subgroup $V\subset F^{\times }$ such that the following properties hold ([10, Proposition 2.8.11 and Definition 2.9.5]):

1. $$H^\times \subset V\,, \quad (F^\times \negthinspace : \negthinspace V) \mid \alpha \,, \quad V(H {\setminus } H^\times ) \subset H$$,
2. $$q^{2\alpha }F \cap H = q^\alpha (q^\alpha F \cap H) \quad \text {for all} \quad q \in F {\setminus } F^\times $$  .

In particular, if $p \in P$ and $a \in p^\alpha F$, then $a \in H$ if and only if $p^\alpha a \in H$.

1. First we suppose that *H* is seminormal. We have to show that ${\mathcal {C}}^*$ is a union of groups. We choose an element $a \in F{\setminus } F^{\times }$, say $a = \epsilon p_1^{k_1}\cdot \ldots \cdot p_t^{k_t}$, where $\epsilon \in F^{\times }$, $p_1,\ldots ,p_t\in P$, $t, k_1,\ldots ,k_t \in {\mathbb {N}}$. Since ${\mathcal {C}}^*$ is finite, there is an $n \in {\mathbb {N}}$ such that $[a^n]$ is an idempotent element of ${\mathcal {C}}^*$. Since for every multiple $n'$ of *n*, $[a^{n'}]$ is an idempotent element of ${\mathcal {C}}^*$, we may assume without restriction that *n* is a multiple of $\alpha$. We assert that $a^{n+1} \sim a$. Clearly, this implies that [*a*] lies in a cyclic subgroup of ${\mathcal {C}}^*$.

In order to show that $a^{n+1} \sim a$, we choose an element $b \in F$. First, suppose that $ab \in H$. Then for every $r\ge \alpha$, we have $(ab)^r\in H\cap p_1^{\alpha }\cdot \ldots \cdot p_t^{\alpha } F$. It follows by Properties (P1) and (P2) that $\epsilon ^n=(\epsilon ^{\alpha })^{\frac{n}{\alpha }}\in V$ and $p_j^{\alpha }(ab)^r\in H{\setminus } H^{\times }$ for all $j\in [1,t]$. Using Property (P2) repeatedly we infer that

$$\begin{aligned} (a^{n+1}b)^r=a^{rn}(ab)^r=\epsilon ^{rn}p_1^{rk_1 (n/\alpha )\alpha }\cdot \ldots \cdot p_t^{rk_t (n/\alpha )\alpha }(ab)^r\in V(H{\setminus } H^{\times })\subset H\,. \end{aligned}$$

Since *H* is seminormal, we obtain $a^{n+1}b\in H$.

Conversely, suppose that $ba^{n+1} \in H$. We have to verify that $ba \in H$. To do so we claim that

3.1 $$\begin{aligned} (ba)^m a^n \in H \quad \text {for all } \ m \in {\mathbb {N}}\,, \end{aligned}$$

and we proceed by induction on *m*. If $m=1$, then this holds by assumption. Suppose the claim holds for $m \in {\mathbb {N}}$. Then, by the induction hypothesis,

$$\begin{aligned} (ba)^{m+1} a^n \sim (ba)^{m+1}a^{2n}= \Big ((ba)^m a^n \Big ) \Big (b a^{n+1} \Big ) \in H \end{aligned}$$

whence $(ba)^{m+1}a^n \in H$. Using (3.1) with $m=n$ we infer that

$$\begin{aligned} (ba)^n =b^na^n\sim b^na^{2n}=(ba)^n a^n \in H \end{aligned}$$

whence $(ba)^n \in H$. Thus we obtain that

$$\begin{aligned} (ba)^{n+1} =(b^{n+1}a)a^n\sim (b^{n+1}a)a^{2n}= (ba)^n \Big (b a^{n+1} \Big ) \in H \end{aligned}$$

whence $(ba)^{n+1} \in H$. This implies that $(ba)^{\ell } \in H$ for all sufficiently large $\ell \in {\mathbb {N}}$. Since *H* is seminormal, it follows that $ba \in H$.

2. Now we suppose that ${\mathcal {C}}^*$ is a union of groups. Then, by (2.2), the class semigroup ${\mathcal {C}} (H,F)$ is a union of groups. In order to show that *H* is seminormal, let $a \in {\mathsf {q}} (H)$ be given such that $a^n\in H$ for all $n\ge N$ for some $N \in {\mathbb {N}}$. Since *F* is factorial, it is seminormal whence $H \subset F$ and ${\mathsf {q}} (H) \subset {\mathsf {q}} (F)$ imply that $a \in F$. If $a\in F^{\times }$, then $a=a^{N+1}(a^{N})^{-1}\in H$. Suppose $a\in F{\setminus } F^{\times }$. Since $[a]\in {\mathcal {C}}^*$ and ${\mathcal {C}}^*$ is finite, we obtain that $\langle [a]\rangle \subset {\mathcal {C}}^*$ is a finite cyclic group. If $N_0 \in {\mathbb {N}}$ is the order of [*a*] in the cyclic group $\langle [a]\rangle \subset {\mathcal {C}}^*$, then $a\sim a^{N_0t+1}$ for all $t\in {\mathbb {N}}$. Since $N_0N+1>N$, we obtain that $a^{N_0N+1}\in H$ and hence $a\in H$.

3. Suppose that *H* is seminormal and set ${\mathsf {E}} ( {\mathcal {C}}^*) = \{e_0, \ldots , e_n\}$, where $n \in {\mathbb {N}}_0$, $e_0= [1]$, and $e_0 + \cdots + e_n = e_n$. For $i \in [0,n]$, we set ${\mathcal {C}}^*_i = {\mathcal {C}}^*_{e_i}$. Since ${\mathcal {C}}^*$ is a union of groups, (2.1) implies that

$$\begin{aligned} {\mathcal {C}}^* = \biguplus _{i=0}^n {\mathcal {C}}^*_{i} \,. \end{aligned}$$

For every $i \in [0,n]$, we have ${\mathcal {C}}^*_i = \{g \in {\mathcal {C}}^* \mid mg = e_i \ \text {for some } \ m \in {\mathbb {N}}\}$ and ${\mathcal {C}}_i^* + C_n^* = C_n^*$.

For the class group of the Krull monoid ${\widehat{H}}$, we have

$$\begin{aligned} {\mathcal {C}} ({\widehat{H}}) = {\mathsf {q}} ({\mathcal {F}} (P))/{\mathsf {q}} (H_0) = {\mathsf {q}} (F)/{\mathsf {q}} ({\widehat{H}}) = {\mathsf {q}} (F)/{\mathsf {q}} (H) \,. \end{aligned}$$

Since $H_0 \hookrightarrow {\mathcal {F}} (P)$ is a divisor theory, the inclusion ${\widehat{H}} = {\widehat{H}}^{\times } \times H_0 \subset F = {\widehat{H}}^{\times } \times {\mathcal {F}} (P)$ is saturated whence ${\widehat{H}} = {\mathsf {q}} ({\widehat{H}}) \cap F = {\mathsf {q}} (H) \cap F$ ([10, Corollary 2.4.3.2]).

3.(a) Let $a\in H$. If $a\in H^{\times }$, then $[a]=[1]$ is an idempotent of ${\mathcal {C}}^*$. Suppose $a\in H{\setminus } H^{\times }$. We have to show that $a\sim a^2$ and to do so we choose some $b\in F$. If $ab\in H$, then obviously $a^2b\in H$. Conversely, suppose that $a^2b\in H$. Since ${\mathcal {C}}^*$ is a finite Clifford semigroup, there exists an $m \in {\mathbb {N}}$ such that $a^{m+1}\sim a$. Then $ab\sim a^{m+1}b=a^{m-1}a^2b\in H$ which implies $ab\in H$.

3.(b) By [10, Proposition 2.8.7.1], the map $\Phi :{\mathcal {C}}^*\rightarrow {\mathcal {C}} (\widehat{H})$ given by $\Phi ([a])=a{\mathsf {q}}(\widehat{H})$ is an epimorphism. Therefore $\Phi _n = \Phi |_{{\mathcal {C}}^*_n} :{\mathcal {C}}^*_n\rightarrow {\mathcal {C}}(\widehat{H})$ is a group homomorphism, and it remains to show that it is bijective.

Let $b\in F{\setminus } F^{\times }$ such that $[b]=e_n$. Since $e_n$ is the identity of ${\mathcal {C}}_n^*$, $\Phi _n (e_n)$ is the identity element of ${\mathcal {C}}(\widehat{H})$ whence $b {\mathsf {q}} ({\widehat{H}}) = \Phi _n (e_n) = {\mathsf {q}} (H)$ and $b \in {\mathsf {q}} (H) \cap F = \widehat{H}$. Then for every $a\in F$ we have $[ba]=[b]+[a]\in {\mathcal {C}}^*_n$ and $\Phi ([ba])=ba{\mathsf {q}}(\widehat{H})=a{\mathsf {q}}(\widehat{H})$. Thus $\Phi _{n}$ is surjective.

Let $a\in F{\setminus } F^{\times }$ with $[a]\in {\mathcal {C}}^*_n$ such that $\Phi _n ([a])$ is the identity element of ${\mathcal {C}}(\widehat{H})$. Then $a {\mathsf {q}} (H) = {\mathsf {q}} (H)$ and $a \in {\mathsf {q}} (H) \cap F = \widehat{H}$ whence there exists $c\in H$ such that $ca\in H$. Thus 3.(a) implies that [*ca*] and [*c*] are idempotents. Since [*a*] and $e_n$ are in ${\mathcal {C}}^*_n$ and $C_i^* + C_n^* = C_n^*$ for all $i \in [0,n]$, it follows that $[ca] = [c]+[a]$ and $[c]+e_n$ are in ${\mathcal {C}}^*_n$. Since $[ca]=[ca]+[ca]$, it follows that $[ca]=e_n$ and similarly $[c]+e_n=e_n$. Thus we obtain that

$$\begin{aligned}{}[a]=[a]+e_n=[a]+[c]+e_n=[ca]+e_n=e_n \,, \end{aligned}$$

which implies that $\Phi _{n}$ is injective. $\square$

In our next remark and also in Sect. 4, we need the concept of monoids of product-one sequences over groups. Let *G* be a finite group and ${\mathcal {F}} (G)$ be the free abelian monoid with basis *G*. An element $S = g_1 \varvec{\cdot }\ldots \varvec{\cdot }g_{\ell } \in {\mathcal {F}} (G)$ is said to be a *product-one sequence* (over *G*) if its terms can be ordered such that their product equals $1_G$, the identity element of the group *G*. The monoid ${\mathcal {B}} (G)$ of all product-one sequences over *G* is a finitely generated C-monoid, and it is a Krull monoid if and only if *G* is abelian ([6, Theorem 3.2]).

### Remark 3.2

 

1. The main statement of Theorem 1.1 was proved first for the monoid of product-one sequences over a finite group. Let *G* be a finite group and $G'$ be its commutator subgroup. Jun Seok Oh showed that ${\mathcal {B}} (G)$ is seminormal if and only if $|G'|\le 2$ if and only if its class semigroup ${\mathcal {C}}^* ( {\mathcal {B}} (G), {\mathcal {F}} (G))$ is a union of groups ([21, Corollary 3.12]).
2. Let $H \subset F$ be a C-monoid with all conventions as in Theorem 1.1. If $a \in H$, then Theorem 1.1.1 implies that the element $[a] \in {\mathcal {C}} (H,F)$ is idempotent. In case $H = {\mathcal {B}} (G)$, the converse holds and this fact was used in the characterization when ${\mathcal {B}} (G)$ is seminormal. However, the converse does not hold true for general C-monoids as the next example shows.
3. Let $F= {\mathcal {F}} (P)$ with $P = \{p_1,p_2\}$ and
  $$\begin{aligned} H = [p_1p_2, p_1^{2k}p_2 \mid k \ge 0] =\{1, p_1p_2\}\cup \{p_1^{2k}p_2\mid k\ge 0\}\cup \{p_1^tp_2^s \mid t\ge 0, s\ge 2\} \subset F \,. \end{aligned}$$

Then $(H \negthinspace : \negthinspace p_1^2)=(H \negthinspace : \negthinspace p_1^4)=H{\setminus }\{1, p_1p_2\}$ which implies that $[p_1^2]=[p_1^4] \in {\mathcal {C}} (H,F)$. Thus $[p_1^2]$ is an idempotent but $p_1^2 \not \in H$.

## A characterization of half-factoriality

Let *H* be a monoid and ${\mathcal {A}} (H)$ the set of atoms (irreducible elements) of *H*. If an element $a \in H$ has a factorization of the form $a=u_1 \cdot \ldots \cdot u_k$, where $k \in {\mathbb {N}}$ and $u_1, \ldots , u_k \in {\mathcal {A}} (H)$, then *k* is called a factorization length of *a*. The set ${\mathsf {L}}_H (a) = {\mathsf {L}} (a)$ of all factorization lengths is called the *set of lengths* of *a*, and for simplicity we set ${\mathsf {L}} (a) = \{0\}$ for $a \in H^{\times }$. If *H* is *v*-noetherian (which holds true of all C-monoids), then every $a \in H$ has a factorization into atoms and all sets of lengths are finite ( [10, Theorem 2.2.9]). For a finite set $L = \{m_0, \ldots , m_k\} \subset {\mathbb {Z}}$, where $k \in {\mathbb {N}}_0$ and $m_0< \cdots < m_k$, $\Delta (L) = \{m_i - m_{i-1} \mid i \in [1,k]\}$ is called the set of distances of *L*. Then

$$\begin{aligned} {\mathcal {L}} (H) = \{{\mathsf {L}} (a) \mid a \in H \} \quad \text {resp.} \quad \Delta (H) = \bigcup _{L \in {\mathcal {L}} (H)} \Delta (L) \end{aligned}$$

is called the *system of sets of lengths* resp. the *set of distances* of *H*. The monoid *H* is *half-factorial* if $\Delta (H)= \emptyset$ (equivalently, $|L|=1$ for all $L \in {\mathcal {L}} (H)$).

Half-factoriality has always been a central topic in factorization theory (e.g., [3–5, 19, 22, 24]). In 1960 Carlitz proved that a ring of integers in an algebraic number field is half-factorial if and only if the class group has at most two elements. This result (which has a simple proof nowadays) carries over to Krull monoids. Indeed, a Krull monoid with class group *G*, that has a prime divisor in each class, is half-factorial if and only if $|G| \le 2$ (the assumption on the distribution of prime divisors is crucial; we refer to [14, Section 5] for background if this assumption fails). We will need this result and the involved machinery in our study of half-factoriality for C-monoids. Thus, we recall that a monoid homomorphism $\theta :H \rightarrow B$ is a *transfer homomorphism* if the following conditions hold :

**(T 1) **: $$B = \theta (H) B^\times $$ and $$\theta ^{-1} (B^\times ) = H^\times $$.
**(T 2) **: If $u \in H$, $b,\,c \in B$ and $\theta (u) = bc$, then there exist $v,\,w \in H$ such that $u = vw$, $\theta (v)B^{\times } = b B^{\times }$ and $\theta (w)B^{\times } = cB^{\times }$.

If $\theta :H \rightarrow B$ is a transfer homomorphism, then ${\mathcal {L}} (H) = {\mathcal {L}} (B)$ ([10, Proposition 3.2.3]) whence *H* is half-factorial if and only if *B* is half-factorial.

All generalizations of Carlitz’s result beyond the setting of Krull monoids have turned out to be surprisingly difficult. We mention two results valid for special classes of C-domains. There is a characterization of half-factoriality for orders in quadratic number fields in number theoretic terms ([10, Theorem 3.7.15]) and for a class of seminormal weakly Krull domains (including seminormal orders in number fields) in algebraic terms involving the *v*-class group and extension properties of prime divisorial ideals (see [11, Theorem 6.2] and [13]).

In this section we establish a characterization of half-factoriality in terms of the class semigroup, that is valid for all seminormal C-monoids and based on Theorem 1.1. Although it is not difficult to show that the set of distances is finite for all C-monoids ([10, Theorem 3.3.4]), a characterization of half-factoriality turns out to be quite involved compared with the simpleness of the result for Krull monoids. But this difference in complexity stems from the fact that the structure of the class semigroup of a C-monoid *H* can be much more intricate than the structure of the class group ${\mathcal {C}} ({\widehat{H}})$ of its complete integral closure. We provide an explicit example of a half-factorial seminormal C-monoid (Example 4.3) and we also refer to the explicit examples of class semigroups given in [20, Section 4].

We introduce our notation which remains valid throughout the rest of this section. Let $H\subset F=F^{\times }\times {\mathcal {F}}(P)$ be a dense seminormal C-monoid with ${\mathsf {E}}({\mathcal {C}}) = \{e_0 = [1], \ldots , e_n\}$, where $n \in {\mathbb {N}}$ and $e_0+ \cdots + e_n = e_n$. For a subset $T \subset F$, we define

$$\begin{aligned} {\mathcal {C}}_T (H,F) = \{ [y] \mid y \in T\} \subset {\mathcal {C}} (H,F) \,. \end{aligned}$$

We use the abbreviations

$$\begin{aligned} {\mathcal {C}}= & {} {\mathcal {C}} (H,F), \ {\mathcal {C}}^* = {\mathcal {C}}^* (H,F), \ {\mathcal {C}}_H = {\mathcal {C}}_H (H,F), \ {\mathcal {C}}_i^*={\mathcal {C}}^*_{e_i}, \quad \text {and} \\&{\mathcal {C}}_i = {\mathcal {C}}_{e_i} \quad \text {for all} \ i \in [0,n] \,, \end{aligned}$$

whence

$$\begin{aligned} {\mathsf {E}} ({\mathcal {C}}^*) = {\mathsf {E}} ({\mathcal {C}}),\ \ {\mathcal {C}}= & {} \biguplus _{i=0}^n {\mathcal {C}}_i, \ {\mathcal {C}}^*=\biguplus _{i=0}^n {\mathcal {C}}_i^*,\ \ {\mathcal {C}}_i={\mathcal {C}}_i^* \ \text {for all} \ i \in [1,n],\ \text { and }\\&{\mathcal {C}}^{\times }={\mathcal {C}}_0={\mathcal {C}}_{F^{\times }}(H,F)\cup {\mathcal {C}}_0^*, \end{aligned}$$

### Proposition 4.1

Let $H\subset F=F^{\times }\times {\mathcal {F}}(P)$ be a dense seminormal C-monoid (with all notations as above) and suppose that every class of ${\mathcal {C}}^*$ contains an element from *P*. For every $i\in [0,n]$, let $P_i=\{p\in P\mid [p]\in {\mathcal {C}}_i^*\}$, $F_i=F^{\times }\times {\mathcal {F}}(P_i)$, $H_i=F_i\cap H$, and let $\varphi _i :{\mathcal {C}}_{F^{\times }}(H,F)\rightarrow {\mathcal {C}}_i^*$ be defined by $\varphi _i([\epsilon ])=[\epsilon ]+e_i$ for all $\epsilon \in F^{\times }$. Then for every $k\in [0,n]$, $H_k \subset F_k$ is a seminormal C-monoid and the following statements hold :

1. If $e_k\not \in {\mathcal {C}}_H$, then $H_k=H^{\times }$. If $e_k\in {\mathcal {C}}_H$, then
  $$\begin{aligned} {\mathcal {C}}(H_k, F_k) \cong {\left\{ \begin{array}{ll} {\mathcal {C}}_k, &{} \text {if } \varphi _k \text { is injective}, \\ {\mathcal {C}}_{F^{\times }}(H,F) \cup {\mathcal {C}}_k, &{} \text {if } \varphi _k \text { is not injective.} \end{array}\right. } \end{aligned}$$
  In particular, ${\mathcal {C}}(H_0,F_0)\cong {\mathcal {C}}_0$ and if ${\mathcal {C}} (H_k, F_k)$ is a group, then $H_k$ is a Krull monoid.
2. If $e_k\in {\mathcal {C}}_H$, then there is a transfer homomorphism $\theta _k :H_k\rightarrow {\mathcal {B}} \big ({\mathcal {C}}_k^*/\varphi _k({\mathcal {C}}_{F^{\times }}(H,F)) \big )$.

### Proof

Let $k\in [0,n]$. By [10, Proposition 2.9.9], $H_k \subset F_k$ is a C-monoid and it is a divisor-closed submonoid of *H*. Since $H_k$ is a divisor-closed submonoid of the seminormal monoid *H*, it is seminormal (this is easy to check; for details see [11, Lemma 3.2]). By [10, Lemma 2.8.4.5], $\psi _k :{\mathcal {C}}_{F_k}(H,F)\rightarrow {\mathcal {C}}(H_k,F_k)$, defined by $\psi _k([a]_H^F)=[a]_{H_k}^{F_k}$ for $a\in F_k$, is an epimorphism. By definition, we have ${\mathcal {C}}_{F_k}(H,F)= {\mathcal {C}}_{F^{\times }}(H,F)\cup {\mathcal {C}}_k^*={\mathcal {C}}_{F^{\times }}(H,F)\cup {\mathcal {C}}_k$.

1. First suppose $e_k\not \in {\mathcal {C}}_H$. It is clear that $H^{\times }\subset H\cap F_k=H_k$. Assume to the contrary that there exists an element $a\in H_k{\setminus } H^{\times }$. If $a\in F^{\times }$, then $a\in F^{\times }\cap H= H^{\times }$, a contradiction. If $a\in F_k{\setminus } F^{\times }$, then $[a]_H^F\in {\mathcal {C}}_k$ and again Theorem 1.1.1 implies that $[a]_H^F=e_k\in {\mathcal {C}}_H$, a contradiction.

Now we suppose that $e_k \in {\mathcal {C}}_H$.

1.(a) Suppose that $\varphi _k$ is injective. We want to show that $\psi _{k}|_{{\mathcal {C}}_k} :{\mathcal {C}}_k\rightarrow {\mathcal {C}}(H_k,F_k)$ is bijective.

First, we show that $\psi _{k}|_{{\mathcal {C}}_k}$ is surjective. Let $p\in P_k$ such that $[p]_H^F=e_k$ and $\epsilon \in F^{\times }$. Then $p\in H_k$. It suffices to prove $[\epsilon ]_{H_k}^{F_k}=[\epsilon p]_{H_k}^{F_k}$.

Let $a\in F_k$. If $\epsilon a\in H_k$, then $\epsilon pa\in H_k$. Suppose $\epsilon pa\in H_k$. Then $[\epsilon pa]_H^F=[\epsilon a]_H^F+e_k=e_k$. If $[\epsilon a]_H^F\in {\mathcal {C}}_k$, then $[\epsilon a]_H^F=e_k$ and hence $\epsilon a\in H\cap F_k= H_k$. Suppose $[\epsilon a]_H^F\in {\mathcal {C}}_{F^{\times }}(H,F)$. Since $\varphi _k$ is injective, we obtain $[\epsilon a]_H^F=[1]_H^F$ whence $\epsilon a\in H_k$.

In order to show that $\psi _{k}|_{{\mathcal {C}}_k}$ is injective, let $p_1, p_2\in P_k$ be given such that $[p_1]_H^F\ne [p_2]_H^F$. Since every class of ${\mathcal {C}}^*$ contains an element from *P*, there is a $p\in P_k$ such that $[p]_H^F=-[p_1]_H^F$. Then $pp_1\in H_k$ and $pp_2\not \in H_k$ which implies that $[p_1]_{H_k}^{F_k}\ne [p_2]_{H_k}^{F_k}$.

1.(b) Suppose that $\varphi _k$ is not injective. It suffices to prove $\psi _k$ is injective. Let $a_1, a_2\in F_k$ such that $[a_1]_H^F\ne [a_2]_H^F$. We have to show that $[a_1]_{H_k}^{F_k}\ne [a_2]_{H_k}^{F_k}$ and to do so we distinguish three cases.

If $[a_1]_H^F, [a_2]_H^F\in {\mathcal {C}}_{F^{\times }}(H,F)$, then there exists an $\epsilon \in F^{\times }$ such that $[\epsilon ]_H^F=-[a_1]_H^F$. Therefore $\epsilon a_1\in H_k$ and $\epsilon a_2\not \in H_k$ which imply $[a_1]_{H_k}^{F_k}\ne [a_2]_{H_k}^{F_k}$.

If $[a_1]_H^F, [a_2]_H^F\in {\mathcal {C}}_k$, then there exists an $a \in F_k$ such that $[a]_H^F=-[a_1]_H^F$. Therefore $a a_1\in H_k$ and $a a_2\not \in H_k$ which imply $[a_1]_{H_k}^{F_k}\ne [a_2]_{H_k}^{F_k}$.

By symmetry, it remains to consider the case where $[a_1]_H^F \in {\mathcal {C}}_k$ and $[a_2]_H^F\in {\mathcal {C}}_{F^{\times }}(H,F)$. Then there exists $\epsilon \in F^{\times }$ such that $[\epsilon ]_H^F=-[a_2]_H^F$. Therefore $\epsilon a_2\in H_k$. Assume to the contrary that $[a_1]_{H_k}^{F_k}=[a_2]_{H_k}^{F_k}$. Then $\epsilon a_1\in H_k$. Since $\varphi _k$ is not injective, there exists $\eta \in F^{\times }$ such that $[\eta ]_H^F\ne [1]_H^F$ and $[\eta ]_H^F+e_k=e_k$. Then $[\eta \epsilon a_1]=e_k$ whence $\eta \epsilon a_1\in H_k$ and $\eta \epsilon a_2\in H_k$. But $[\eta \epsilon a_2]_H^F=[\eta ]_H^F\ne [1]_H^F$, a contradiction.

1.(c) Since ${\mathcal {C}}_{F^{\times }}(H,F)\cup {\mathcal {C}}_0={\mathcal {C}}_0$, we have ${\mathcal {C}}(H_0,F_0)\cong {\mathcal {C}}_0$. If ${\mathcal {C}} (H_k, F_k)$ is a group, then $H_k$ is a Krull monoid by (2.3) and (2.5).

2. Suppose that $e_k \in {\mathcal {C}}_H$. For $g\in {\mathcal {C}}_k^*$, we set ${\overline{g}}=g+\varphi _k( {\mathcal {C}}_{F^{\times }}(H,F))\in {\mathcal {C}}_k^*/\varphi _k( {\mathcal {C}}_{F^{\times }}(H,F))$ and we define

$$\begin{aligned} \begin{aligned} \theta ^* :F_k&\ \longrightarrow \ {\mathcal {F}}({\mathcal {C}}_k^*/\varphi _k( {\mathcal {C}}_{F^{\times }}(H,F))) \\ a=\epsilon \prod _{p\in P_k}p^{{\mathsf {v}}_p(a)}&\ \longmapsto \ \prod _{p\in P_k}\overline{[p]_H^F}^{{\mathsf {v}}_p(a)} \,. \end{aligned} \end{aligned}$$

First, we show that $\theta ^*(H_k)={\mathcal {B}}({\mathcal {C}}_k^*/\varphi _k( {\mathcal {C}}_{F^{\times }}(H,F)))$. Note $H_k=H\cap F_k=\big \{x\in F_k\mid [x]_H^F\in \{e_0, e_k\}\big \}$. If $a\in H_k^{\times }$, then $\theta ^*(a)=1_{{\mathcal {F}}({\mathcal {C}}_k^*/\varphi _k( {\mathcal {C}}_{F^{\times }}(H,F)))}$. If $a=\epsilon \prod _{p\in P_k}p^{{\mathsf {v}}_p(a)}\in H_k{\setminus } H_k^{\times }$, then the sum of the elements of $\theta ^*(a)$ equals $\sum _{p\in P_k}{\mathsf {v}}_p(a)\overline{[p]_H^F} = \overline{[a]_H^F}=\overline{e_k}$, which is the zero element of the group ${\mathcal {C}}_k^*/\varphi _k( {\mathcal {C}}_{F^{\times }}(H,F))$. Thus we obtain that $\theta ^*(H_k)\subset {\mathcal {B}}({\mathcal {C}}_k^*/\varphi _k( {\mathcal {C}}_{F^{\times }}(H,F)))$ and in order to verify equality, we choose an $S=\overline{g_1} \cdot \ldots \cdot \overline{g_{\ell }}\in {\mathcal {B}}({\mathcal {C}}_k^*/\varphi _k( {\mathcal {C}}_{F^{\times }}(H,F)))$, where $g_1,\ldots , g_{\ell }\in {\mathcal {C}}_k^*$. By assumption, there exist $p_1,\ldots ,p_{\ell }\in P_k$ such that $[p_i]_H^F=g_i$ for all $i\in [1,\ell ]$. Since

$$\begin{aligned} \overline{[p_1 \cdot \ldots \cdot p_{\ell }]_H^F} = \overline{[p_1]_H^F} + \cdots + \overline{[p_{\ell }]_H^F} = \overline{g_1} + \cdots + \overline{g_{\ell }} = 0_{{\mathcal {C}}_k^*/\varphi _k({\mathcal {C}}_{F^{\times }}(H,F))} \,, \end{aligned}$$

we infer that $[p_1 \cdot \ldots \cdot p_{\ell }]_H^F\in \varphi _k( {\mathcal {C}}_{F^{\times }}(H,F))$. Thus there is an $\epsilon \in F^{\times }$ such that $\varphi _k([\epsilon ]_H^F)=-[p_1 \cdot \ldots \cdot p_{\ell }]_H^F$. So it follows that $[\epsilon p_1 \cdot \ldots \cdot p_{\ell }]_H^F = e_k$ whence $\epsilon p_1 \cdot \ldots \cdot p_{\ell }\in H\cap F_k=H_k$ and $\theta ^*(\epsilon p_1 \cdot \ldots \cdot p_{\ell })=S$.

Secondly, we show $\theta =\theta ^*|_{H_k}:H_k\rightarrow {\mathcal {B}}({\mathcal {C}}_k^*/\varphi _k( {\mathcal {C}}_{F^{\times }}(H,F)))$ is a transfer homomorphism. Clearly, ${\mathcal {B}} (\cdot )$ is reduced and **(T1)** holds. In order to verify **(T2)**, let $a\in H_k$ and $b,c\in {\mathcal {B}}({\mathcal {C}}_k^*/\varphi _k( {\mathcal {C}}_{F^{\times }}(H,F))) {\setminus } \{1\}$ such that $\theta (a)=bc$. We set $a=\epsilon p_1 \cdot \ldots \cdot p_{\ell }$, where $\epsilon \in F^{\times }$, $\ell \in {\mathbb {N}}_0$, $p_1,\ldots , p_{\ell }\in P_k$ and, after renumbering if necessary, we assume $b=\overline{[p_1]_H^F} \cdot \ldots \cdot \overline{[p_s]_H^F}$ and $c=\overline{[p_{s+1}]_H^F} \cdot \ldots \cdot \overline{[p_{\ell }]_H^F}$, where $s\in [1,\ell -1]$. Thus there exists an $\epsilon _1\in F^{\times }$ such that $\varphi _k ([\epsilon _1]_H^F)= [\epsilon _1]_H^F + e_k =-[p_1 \cdot \ldots \cdot p_s]_H^F$ and hence

$$\begin{aligned}{}[\epsilon \epsilon _1^{-1}]_H^F + e_k= & {} [\epsilon ]_H^F + e_k -[\epsilon _1]_H^F + e_k =-[p_1 \cdot \ldots \cdot p_{\ell }]_H^F+[p_1 \cdot \ldots \cdot p_s]_H^F\\= & {} -[p_{s+1} \cdot \ldots \cdot p_{\ell }]_H^F. \end{aligned}$$

It follows that $u=\epsilon _1 p_1 \cdot \ldots \cdot p_s\in H_k$, $v=\epsilon \epsilon _1^{-1}p_{s+1} \cdot \ldots \cdot p_{\ell }\in H_k$, $a=uv$, $\theta (u)=b$, and $\theta (v)=c$. $\square$

### Theorem 4.2

Let $H\subset F=F^{\times }\times {\mathcal {F}}(P)$ be a dense seminormal C-monoid (with all notations as above) and suppose that every class of ${\mathcal {C}}^*$ contains an element from *P*. For every $i\in [0,n]$, let $\varphi _i :{\mathcal {C}}_{F^{\times }}(H,F)\rightarrow {\mathcal {C}}_i^*$ be defined by $\varphi _i([\epsilon ])=[\epsilon ]+e_i$ for all $\epsilon \in F^{\times }$ and set ${\mathcal {C}}_i'={\mathcal {C}}_i^*/\varphi _i( {\mathcal {C}}_{F^{\times }}(H,F))$. For distinct $i,j\in [0,n]$, let $\phi _{i,j} :{\mathcal {C}}_i^*\rightarrow {\mathcal {C}}_i^*+{\mathcal {C}}_j^*$ be defined by $\phi _{i,j}(g_i)=g_i+e_j$ for all $g_i\in {\mathcal {C}}_i^*$.

Then *H* is half-factorial if and only if the following properties hold :

1. $$|{\mathcal {C}}_n'|\le 2$$.
2. For every $i\in [0,n]$, we have
  $$\begin{aligned} \phi _{i,n}^{-1}(\varphi _n({\mathcal {C}}_{F^{\times }}(H,F)))=\left\{ \begin{aligned}&\varphi _i({\mathcal {C}}_{F^{\times }}(H,F)),&\quad \quad e_i\in {\mathcal {C}}_H\\&{\mathcal {C}}_i,&\qquad e_i\not \in {\mathcal {C}}_H \end{aligned} \right. \end{aligned}$$
3. For distinct $i,j\in [0,n]$, if $e_i,e_j\in {\mathcal {C}}_H$, then $\ker (\phi _{i,j} \circ \varphi _i)=\ker (\varphi _i)+ \ker (\varphi _j)$.
4. ${\mathsf {E}}({\mathcal {C}}^*){\setminus } {\mathcal {C}}_H$ is additively closed and for distinct $i_1,i_2, j\in [0,n]$, if $e_{i_1}, e_{i_2}\in {\mathcal {C}}_H$ and $e_j\in {\mathsf {E}}({\mathcal {C}}^*){\setminus } {\mathcal {C}}_H$ such that $e_{i_1}+e_{i_2}+e_j\in {\mathcal {C}}_H$, then $e_{i_1}+e_j\in {\mathcal {C}}_H$ or $e_{i_2}+e_j\in {\mathcal {C}}_H$.

### Proof

In order to show that $e_n\in {\mathcal {C}}_H$, we choose a $p^* \in P$ such that $[p^*]=e_n$. Since $H\subset F$ is dense, there exists an $a\in H$ such that $p^* \mid a$, say $a=p^*b$ with $b \in F$. By Theorem 1.1, [*a*] is an idempotent and since $[a] = [p^*]+[b]=e_n+[b] \in {\mathcal {C}}_n$, it follows that $e_n = [a] \in {\mathcal {C}}_H$.

**1.** We suppose that *H* is half-factorial and verify properties (P1) - (P4).

1. Let $P_n=\{p\in P\mid [p]\in {\mathcal {C}}_n^*\}$, $F_n=F^{\times }\times {\mathcal {F}}(P_n)$, and $H_n=H\cap F_n$. Since $H_n$ is a divisor closed submonoid of *H*, $H_n$ is half-factorial. By Proposition 4.1.3, there is a transfer homomorphism $\theta :H_n \rightarrow {\mathcal {B}} (C_n')$. Thus ${\mathcal {B}} (C_n')$ is half-factorial which implies that $|{\mathcal {C}}_n'|\le 2$ ([10, Theorem 3.4.11.5]).
2. Let $$i\in [0,n]$$ and $$e_i\in {\mathcal {C}}_H$$. Clearly, $$ e_i+e_n=e_n$$ and $$\phi _{i,n}( {\mathcal {C}}_{F^{\times }}(H,F)+e_i)= {\mathcal {C}}_{F^{\times }}(H,F)+e_n$$. Assume to the contrary that there exists $$g_i\in {\mathcal {C}}_i^*$$ with $$g_i\not \in \varphi _i( {\mathcal {C}}_{F^{\times }}(H,F))$$ such that $$\phi _{i,n}(g_i)\in {\mathcal {C}}_{F^{\times }}(H,F)+e_n$$, i.e. there exists $$\epsilon \in F^{\times }$$ such that $$g_i+e_n=[\epsilon ]+e_n$$. Let $$p_1, p_2, q\in P$$ such that $$[p_1]=g_i$$, $$[p_2]=-g_i$$, and $$[q]=e_n$$. Then $$[\epsilon ^{-1}p_1q]=[\epsilon p_2q]=[q]=e_n$$ and $$[p_1p_2]=e_i$$. We claim that $$\epsilon ^{-1}p_1q, \epsilon p_2q, p_1p_2, q \in {\mathcal {A}} (H)$$. Clearly, the elements lie in *H* and, for example, assume to the contrary that $$p_1p_2$$ is not an atom. Then there is an $$\eta \in F^{\times }$$ such that $$\eta p_1, \eta ^{-1}p_2 \in H$$. Then $$[\eta p_1] = [\eta ] + [p_1] = e_i$$ whence $$g_i = [p_1] = [\eta ^{-1}]+e_i \in \varphi _i \big ({\mathcal {C}}_{F^{\times }} (H,F) \big )$$, a contradiction. Thus we obtain that $$(p_1p_2)(q)^{2}=(\epsilon ^{-1}p_1q)(\epsilon p_2q)$$, a contradiction to the half-factoriality of *H*.
  Suppose $e_i\in {\mathsf {E}}({\mathcal {C}}^*){\setminus } {\mathcal {C}}_H$. Assume to the contrary that there exists $g_i\in {\mathcal {C}}_i$ such that $\phi _{i,n}(g_i)=g_i+e_n\not \in {\mathcal {C}}_{F^{\times }}(H,F)+ e_n$. Let $p_1,p_2,p_3\in P$ such that $[p_1]=g_i$, $[p_2]=e_n$, and $[p_3]=-g_i+e_n$, and let ${{\,\mathrm{ord}\,}}(g_i) = {{\,\mathrm{ord}\,}}_{{\mathcal {C}}_i} (g_i)$ denote the order of $g_i$ in the group ${\mathcal {C}}_i$. Then $[p_1^{{{\,\mathrm{ord}\,}}(g_i)}p_2]=[p_2]=[p_1p_3]=e_n\in {\mathcal {C}}_H$ which implies that $p_1^{{{\,\mathrm{ord}\,}}(g_i)}p_2, p_2, p_1p_3 \in {\mathcal {A}} (H)$. Therefore $(p_1p_3)^{{{\,\mathrm{ord}\,}}(g_i)}p_2=(p_1^{{{\,\mathrm{ord}\,}}(g_i)}p_2)(p_3^{{{\,\mathrm{ord}\,}}(g_i)})$. Since $p_3^{{{\,\mathrm{ord}\,}}(g_i)}\in H$ and for any $\epsilon \in F^{\times }$, $\epsilon p_3\not \in H$, we obtain ${{\,\mathrm{ord}\,}}(g_i)\not \in {\mathsf {L}}_H (p_3^{{{\,\mathrm{ord}\,}}(g_i)})$, a contradiction to the half-factoriality of *H*.
3. Let $i,j\in [0,n]$ be distinct and $e_i,e_j\in {\mathcal {C}}_H$. Since $\phi _{i,j} \circ \varphi _i =\phi _{j,i} \circ \varphi _j$, we have $\ker (\phi _{i,j} \circ \varphi _i) \supset \ker (\varphi _i)$ and $\ker (\phi _{i,j} \circ \varphi _i)\supset \ker (\varphi _j)$ which imply $\ker (\phi _{i,j} \circ \varphi _i )\supset \ker (\varphi _i)+\ker (\varphi _j)$. Let $\epsilon \in F^{\times }$ such that $\phi _{i,j}(\varphi _i([\epsilon ]))=e_i+e_j$. If $[\epsilon ]+e_i=e_i$ or $[\epsilon ]+e_j=e_j$, then $[\epsilon ] = [\epsilon ]+[1] \in \ker (\varphi _i)+\ker (\varphi _j)$. Suppose $[\epsilon ]+e_i\ne e_i$ and $[\epsilon ]+e_j\ne e_j$. Let $p_1, p_2, p_3\in P$ such that $[p_1]=[\epsilon ]+e_i$, $[p_2]=e_j$, and $[p_3]=e_i+e_j$. Since $p_2, p_3, \epsilon p_3, \epsilon ^{-1}p_1 \in {\mathcal {A}} (H)$, the equation $(p_1p_2)p_3=(\epsilon ^{-1}p_1)(\epsilon p_3)p_2$ implies that $p_1p_2 \notin {\mathcal {A}} (H)$. Then there exists $\delta \in F^{\times }$ such that $\delta p_1 \in H$ and $\delta ^{-1}p_2 \in H$ whence $[\delta p_1]= [\delta ]+[\epsilon ]+e_i=e_i$ and $[\delta ^{-1}p_2]= -[\delta ]+e_j=e_j$. It follows that $[\delta ]+[\epsilon ]\in \ker (\varphi _i)$, $-[\delta ]\in \ker (\varphi _j)$, and $[\epsilon ]=[\delta ]+[\epsilon ]-[\delta ]\in \ker (\varphi _i)+\ker (\varphi _j)$.
4. Assume to the contrary that there exist $f_1,f_2 \in {\mathsf {E}}({\mathcal {C}}^*){\setminus } {\mathcal {C}}_H$ such that $f_1+f_2\in {\mathcal {C}}_H$. Let $q_1,q_2\in P$ such that $[q_1]=f_1$ and $[q_2]=f_2$. Then $[q_1q_2]=[q_1^2q_2]=[q_1q_2^2]=f_1+f_2\in {\mathcal {C}}_H$, $q_1q_2, q_1^2q_2, q_1q_2^2\in {\mathcal {A}} (H)$, and $(q_1q_2)^3= (q_1^2q_2) (q_1q_2^2)$, a contradiction to the half-factoriality of *H*.
  Let $i_1,i_2, j\in [0,n]$ such that $e_j\in {\mathsf {E}}({\mathcal {C}}^*){\setminus } {\mathcal {C}}_H$ and $e_{i_1}, e_{i_2}, e_{i_1}+e_{i_2}+e_j\in {\mathcal {C}}_H$. Assume to the contrary that $e_{i_1}+e_j, e_{i_2}+e_j\in {\mathsf {E}}({\mathcal {C}}^*){\setminus } {\mathcal {C}}_H$. Let $p,p_1,p_2,p_3\in P$ such that $[p]=e_{i_1}+e_{i_2},[p_1]=e_{i_1}, [p_2]=e_{i_2}, [p_3]=e_j$. Then $p, p_1, p_2,pp_3, p_1p_2p_3 \in {\mathcal {A}} (H)$ and $(p)(p_1p_2p_3)=(p_1)(p_2)(pp_3)$, a contradiction to the half-factoriality of *H*.

**2.** We suppose that (P1) - (P4) hold and prove that *H* is half-factorial. We start with the following three assertions.

**A1.** : If $i \in [0,n]$ and $e_i\in {\mathcal {C}}_H$, then $|{\mathcal {C}}_i'|\le 2$.
**A2.** : If $i,j \in [0,n]$ and $e_i,e_j\in {\mathcal {C}}_H$, then $\phi _{i,j}^{-1}( {\mathcal {C}}_{F^{\times }}(H,F)+e_i+e_j)= {\mathcal {C}}_{F^{\times }}(H,F)+e_i$.
**A3.** : If $i,j \in [0,n]$, $e_i\not \in {\mathcal {C}}_H$, and $e_j\in {\mathcal {C}}_H$ such that $e_i+e_j\in {\mathcal {C}}_H$, then $\phi _{i,j}^{-1}( {\mathcal {C}}_{F^{\times }}(H,F)+e_i+e_j)={\mathcal {C}}_i$.

*Proof of  ***A1** .  Let $i \in [0,n]$ and $e_i\in {\mathcal {C}}_H$. By (P2), there is a monomorphism $\phi _{i,n}' :{\mathcal {C}}_i'\rightarrow {\mathcal {C}}_n'$ defined by $\phi _{i,n}'(h+\varphi _i( {\mathcal {C}}_{F^{\times }}(H,F)))=h+e_n+\varphi _n( {\mathcal {C}}_{F^{\times }}(H,F))$ for all $h\in {\mathcal {C}}_i^*$. It follows by (P1) that $|{\mathcal {C}}_i'|\le 2$.

*Proof of  ***A2** .  Let $i,j \in [0,n]$ and $e_i,e_j\in {\mathcal {C}}_H$, say $e_i+e_j=e_k$ with $k \in [0,n]$, and note that $\phi _{i,n}=\phi _{k,n} \circ \phi _{i,k}$ and $\phi _{i,j}=\phi _{i,k}$. Then (P2) implies that ${\mathcal {C}}_{F^{\times }}(H,F)+e_i=\phi _{i,n}^{-1}( {\mathcal {C}}_{F^{\times }}(H,F)+e_n)=\phi _{i,k}^{-1}(\phi _{k,n}^{-1}( {\mathcal {C}}_{F^{\times }}(H,F)+e_n))=\phi _{i,j}^{-1}( {\mathcal {C}}_{F^{\times }}(H,F)+e_k)$.

*Proof of  ***A3** .  Let $i,j \in [0,n]$, $e_i\in {\mathsf {E}}({\mathcal {C}}^*){\setminus } {\mathcal {C}}_H$, and $e_j\in {\mathcal {C}}_H$ such that $e_i+e_j \in {\mathcal {C}}_H$, say $e_i+e_j=e_k$ with $k \in [0,n]$, and again we note that $\phi _{i,n}=\phi _{k,n} \circ \phi _{i,k}$ and $\phi _{i,j}=\phi _{i,k}$. Then (P2) implies that ${\mathcal {C}}_i=\phi _{i,n}^{-1}( {\mathcal {C}}_{F^{\times }}(H,F)+e_n)=\phi _{i,k}^{-1}(\phi _{k,n}^{-1}( {\mathcal {C}}_{F^{\times }}(H,F)+e_n))=\phi _{i,j}^{-1}( {\mathcal {C}}_{F^{\times }}(H,F)+e_k)$.

Now we set $I=\{i\in [0,n]\mid e_i\in {\mathcal {C}}_H\}$ and we define

$$\begin{aligned} G^1=\bigcup _{i\in I} \varphi _i \big ({\mathcal {C}}_{F^{\times }}(H,F) \big ), \ G^2=\bigcup _{i\in I} \big ({\mathcal {C}}_i^*{\setminus } \varphi _i({\mathcal {C}}_{F^{\times }}(H,F)) \big ), \ \text { and } \ G^3={\mathcal {C}}^*{\setminus } \bigcup _{i\in I}{\mathcal {C}}_i^* \,. \end{aligned}$$

For every $a=\epsilon p_1 \cdot \ldots \cdot p_{\ell }\in F$, where $\epsilon \in F^{\times }$, $\ell \in {\mathbb {N}}_0$, and $p_1,\ldots , p_{\ell }\in P$, we define

$$\begin{aligned} {\mathsf {l}}_1(a)= & {} |\{j\in [1,\ell ]\mid [p_j]\in G^1\}|\,,\ {\mathsf {l}}_2(a) = |\{k\in [1,\ell ]\mid [p_k]\in G^2\}|\,, \quad \text {and } \\&{\mathsf {l}}(a)={\mathsf {l}}_1(a)+\frac{1}{2}{\mathsf {l}}_2(a)\,. \end{aligned}$$

In order to prove that *H* is half-factorial, it is sufficient to prove the following assertion.

**A4. **If *a* is an atom of *H*, then ${\mathsf {l}}(a)=1$.

*Proof of  ***A4** .  Let $a=\epsilon p_1 \cdot \ldots \cdot p_{\ell }\in F{\setminus } F^{\times }$ be an atom of *H*. Assume to the contrary that ${\mathsf {l}}(a)=0$. Then, for every $i\in [1,\ell ]$, we have $[p_i]\in G^3$, say $[p_i] \in {\mathcal {C}}_{f_i}$ with $f_i\in {\mathsf {E}}({\mathcal {C}}^*){\setminus } {\mathcal {C}}_H$. Clearly, we have $[a]=[\epsilon ]+[p_1]+\cdots +[p_{\ell }] \in {\mathcal {C}}_{[a]}$ and $[a]\in {\mathcal {C}}_{F^{\times }}(H, F) + {\mathcal {C}}_{f_1} + \cdots + {\mathcal {C}}_{f_{\ell }} \subset {\mathcal {C}}_{f_1+\cdots +f_{\ell }}$ whence $f_1+\cdots +f_{\ell }=[a]$, a contradiction to (P4).

Assume to the contrary that ${\mathsf {l}}(a)\ge 2$ and distinguish three cases.

CASE 1: $${\mathsf {l}}_1(a)\ge 2$$.

After renumbering if necessary, we may assume that $[p_1],[p_2]\in G^1$. Then there exists $\epsilon _1,\epsilon _2\in F^{\times }$ and $e_i,e_j\in {\mathcal {C}}_H$ such that $[p_1]=[\epsilon _1]+e_i, [p_2]=[\epsilon _2]+e_j$. We set $g=[\epsilon p_3 \cdot \ldots \cdot p_{\ell }]\in {\mathcal {C}}_k$ and $[a]=e_{r}\in {\mathcal {C}}_H$ for some $k,r\in [0,n]$. Then

$$\begin{aligned} e_r = [\epsilon _1] + e_i + [\epsilon _2]+e_j + g \in {\mathcal {C}}_{F^{\times }} (H,F)+ {\mathcal {C}}_i + {\mathcal {C}}_j + {\mathcal {C}}_k \subset {\mathcal {C}}_{e_i+e_j+e_k} \end{aligned}$$

whence $e_i+e_j+e_k=e_{r}\in {\mathcal {C}}_H$. By (P4), we obtain that $e_i+e_k\in {\mathcal {C}}_H$ or $e_j+e_k\in {\mathcal {C}}_H$, say $e_j+e_k=e_t\in {\mathcal {C}}_H$ for some $t\in [0,n]$.

Since $$[\epsilon _1]+[p_2]+g\in {\mathcal {C}}_t$$ and

$$\begin{aligned} \phi _{t,i}([\epsilon _1]+[p_2]+g)= & {} [\epsilon _1] + [p_2]+g+e_i = [p_1]+[p_2]+g= [\epsilon p_1 \cdot \ldots \cdot p_{\ell }]=e_r \\= & {} e_i+e_t \in {\mathcal {C}}_{F^{\times }}(H,F)+e_i+e_t, \end{aligned}$$

we obtain $[\epsilon _1]+[p_2]+g\in {\mathcal {C}}_{F^{\times }}(H,F)+e_t$ by **A2**. It follows that $[\epsilon p_2 \cdot \ldots \cdot p_{\ell }]=[p_2]+g\in {\mathcal {C}}_{F^{\times }}(H,F)+e_t$ whence there exists $\epsilon _3\in F^{\times }$ such that $[\epsilon p_2 \cdot \ldots \cdot p_{\ell }]=[\epsilon _3]+e_t$. Since

$$\begin{aligned} e_i+e_t=e_r = [a]=[p_1]+[\epsilon p_2 \cdot \ldots \cdot p_{\ell }]= [\epsilon _1]+e_i+[\epsilon _3]+e_t \,, \end{aligned}$$

we infer that $[\epsilon _1]+[\epsilon _3]\in \ker (\phi _{i,t} \circ \varphi _i )$. By (P3), there exists $\delta \in F^{\times }$ such that $[\delta ]\in \ker (\varphi _i)$ and $[\epsilon _1\epsilon _3\delta ^{-1}]\in \ker (\varphi _t)$. Thus we obtain that

$$\begin{aligned}&[\delta \epsilon _1^{-1}p_1]=[\delta ]-[\epsilon _1]+[p_1]=[\delta ]+e_i=e_i\\&[\epsilon _1\delta ^{-1}\epsilon p_2 \cdot \ldots \cdot p_{\ell }]=[\epsilon _1\epsilon _3\delta ^{-1} \epsilon _3^{-1} \epsilon p_2 \cdot \ldots \cdot p_{\ell }]=[\epsilon _1\epsilon _3\delta ^{-1}]-[\epsilon _3]\\&\quad +[\epsilon p_2 \cdot \ldots \cdot p_{\ell }]=[\epsilon _1\epsilon _2\delta ^{-1}]+e_t=e_t\,. \end{aligned}$$

Thus $\delta \epsilon _1^{-1}p_1 \in H {\setminus } H^{\times }$, $\epsilon _1\delta ^{-1}\epsilon p_2 \cdot \ldots \cdot p_{\ell } \in H {\setminus } H^{\times }$, and $a=(\delta \epsilon _1^{-1}p_1)(\epsilon _1\delta ^{-1}\epsilon p_2 \cdot \ldots \cdot p_{\ell })$, a contradiction to $a \in {\mathcal {A}} (H)$.

CASE 2: $${\mathsf {l}}_1(a)=1$$ and $${\mathsf {l}}_2(a)\ge 2$$.

After renumbering if necessary, we may assume that $[p_1]\in G^1$, and $[p_2], [p_3]\in G^2$. There are $i,j, k\in [0,n]$ such that $[p_2]\in {\mathcal {C}}_i^* {\setminus } \varphi _i ({\mathcal {C}}_{F^{\times }} (H,F)$, $[p_3]\in {\mathcal {C}}_j^* {\setminus } \varphi _j ({\mathcal {C}}_{F^{\times }} (H,F)$, and $e_k=e_i+e_j$. By **A2** we infer that

$$\begin{aligned} \phi _{i,k}([p_2])=[p_2]+e_k\not \in {\mathcal {C}}_{F^{\times }}(H,F)+e_i+e_k=\varphi _k( {\mathcal {C}}_{F^{\times }}(H,F)) \end{aligned}$$

and

$$\begin{aligned} \phi _{i,k}([p_3])=[p_3]+e_k\not \in {\mathcal {C}}_{F^{\times }}(H,F)+e_i+e_k=\varphi _k( {\mathcal {C}}_{F^{\times }}(H,F)) \,. \end{aligned}$$

Since $[p_2]+e_k$ and $[p_3]+e_k$ are in ${\mathcal {C}}_k^* {\setminus } \varphi _k ({\mathcal {C}}_{F^{\times }} (H,F))$ and since, by **A1**, $|{\mathcal {C}}_k'| \le 2$, it follows that $[p_2]+[p_3]= ([p_2]+e_k)+([p_3]+e_k) \in \varphi _k ({\mathcal {C}}_{F^{\times }} (H,F)) \subset G^1$. We choose a $q\in P$ such that $[q]=[p_2]+[p_3]$. Then $b=\epsilon p_1qp_4 \cdot \ldots \cdot p_{\ell } \in {\mathcal {A}} (H)$ with ${\mathsf {l}}(b)={\mathsf {l}}(a)\ge 2$. Now we are back to CASE 1.

CASE 3: $${\mathsf {l}}_1(a)=0$$ and $${\mathsf {l}}_2(a)\ge 4$$.

After renumbering if necessary, we may assume that $[p_1], [p_2], [p_3], [p_4]\in G^2$. Arguing as in CASE 2 we infer that $[p_1]+[p_2], [p_3]+[p_4]\in G^1$. Let $q_1,q_2\in P$ such that $[q_1]=[p_1]+[p_2]$ and $[q_2]=[p_3]+[p_4]$. Then $b=\epsilon q_1q_2p_4 \cdot \ldots \cdot p_{\ell } \in {\mathcal {A}} (H)$ with ${\mathsf {l}}(b)={\mathsf {l}}(a)\ge 2$ whence we are back to CASE 1. $\square$

The following example shows that both the number and the size of the constituent groups of the reduced class semigroup of a half-factorial seminormal C-monoid can be arbitrarily large.

### Example 4.3

Let *C* be a Clifford semigroup with

$$\begin{aligned} {\mathsf {E}}(C)=\{e_0,e_1,\ldots , e_n\}, \ C=\biguplus _{i=0}^n C_i , \quad \text { and } \quad C_{e_i} = C_i = G_i\oplus {\mathbb {Z}}/2 {\mathbb {Z}}\,, \end{aligned}$$

where $n \in {\mathbb {N}}$, $i,j \in [0,n]$, $C_i+e_j=C_j$ whenever $i<j$, and $G_0\supsetneq \cdots \supsetneq G_n=\{1\}$ are abelian groups. Let

$$\begin{aligned} F = F^{\times } \times {\mathcal {F}} ({\mathcal {C}}) \quad \text {and} \quad B=\{\epsilon S\in F \mid \iota (\epsilon )+\sigma (S)\in {\mathsf {E}}(C)\} \,, \end{aligned}$$

where $F^{\times } = G_0$, $\iota :F^{\times }\rightarrow C_0$ is a monomorphism, and $\sigma :{\mathcal {F}} ({\mathcal {C}}) \rightarrow {\mathcal {C}}$ is the sum function, which is defined as $\sigma (g_1 \cdot \ldots \cdot g_{\ell }) = g_1 + \cdots + g_{\ell }$. Then $B\subset F$ is a half-factorial dense seminormal C-monoid such that every class of ${\mathcal {C}}(B,F)$ contains an element from *C*,

$$\begin{aligned} {\mathcal {C}}^*(B,F)={\mathcal {C}}(B,F) \cong C, \ \ {\mathcal {C}}_{F^{\times }}(B,F)\cong F^{\times }, \quad \text {and} \quad {\mathcal {C}}_B(B,F)={\mathsf {E}} \big ({\mathcal {C}}^* (B,F) \big ) \,. \end{aligned}$$

### Proof

We start with the following four assertions. Let $\epsilon , \epsilon _1, \epsilon _2 \in F^{\times }$, $S, S_1, S_2 \in {\mathcal {F}} ({\mathcal {C}})$, and consider the epimorphism

$$\begin{aligned} \lambda :F^{\times }\times {\mathcal {F}}(C)\rightarrow C \text { defined by } \lambda (\epsilon S)=\iota (\epsilon )+\sigma (S) \text { for all }\epsilon S\in F^{\times }\times {\mathcal {F}}(C)\,. \end{aligned}$$

**A1.** : $\epsilon _1S_1\sim \epsilon _2 S_2$ if and only if $\lambda (\epsilon _1 S_1)=\lambda (\epsilon _2 S_2)$. In particular, $[\epsilon S] = [\lambda (\epsilon S)]$.
**A2.** : The map $\psi :{\mathcal {C}}^* (B,F) \rightarrow {\mathcal {C}}$, defined by $[\epsilon S] \mapsto \lambda (\epsilon S)$, is a semigroup isomorphism.
**A3.** : $${\mathcal {C}}_{F^{\times }}(B,F)\cong F^{\times }$$ and $${\mathcal {C}}^*(B,F)={\mathcal {C}}(B,F)$$.
**A4.** : $${\mathcal {C}}_B(B,F)={\mathsf {E}} \big ( {\mathcal {C}}^* (B,F) \big )$$.

Suppose these four statements hold true. Since $B^{\times } = \{1\} = F^{\times } \cap B$, **A2** implies that $B \subset F$ is a C-monoid. Clearly, $B \subset F$ is dense and by Theorem 1.1*B* is seminormal. Since every class of ${\mathcal {C}}^* (B,F)$ contains a prime from *C*, all assumptions of Theorem 4.2 are satisfied and we obtain that *H* is half-factorial.

*Proof of  ***A1** .  By definition of *B*, $\lambda (\epsilon _1 S_1)=\lambda (\epsilon _2 S_2)$ implies that $\epsilon _1S_1\sim \epsilon _2 S_2$. Conversely, suppose $\epsilon _1S_1\sim \epsilon _2 S_2$ and $\lambda (\epsilon _1 S_1)\ne \lambda (\epsilon _2 S_2)$. If there exists an $i\in [0,n]$ such that $\lambda (\epsilon _1 S_1),\lambda (\epsilon _2 S_2)\in C_i$, then $a=-\lambda (\epsilon _1 S_1)\in {\mathcal {C}} \subset F$ and hence $\epsilon _1 S_1 a\in B$, $\epsilon _2 S_2 a\not \in B$, a contradiction. After renumbering if necessary we may assume that $\lambda (\epsilon _1 S_1)\in C_i,\lambda (\epsilon _2 S_2)\in C_j$ for some $i,j\in [0,n]$ with $i<j$. Let $a=-\lambda (\epsilon _1 S_1)\in {\mathcal {C}} \subset F$. Since $G_i\supsetneq G_j$ and $C_i+e_j=C_j$, there exists $g_i\in C_i{\setminus } \{e_i\}$ such that $g_i+e_j=e_j$. Then $\epsilon _1 S_1 a\in B$ which implies that $\epsilon _2 S_2 a\in B$. Thus $\epsilon _2 S_2 a g_i\in B$ which implies that $\epsilon _1 S_1 a g_i\in B$. But $\lambda (\epsilon _1 S_1 a g_i)=g_i\ne e_i$, a contradiction.

To verify the in particular statement, note that $\lambda (\epsilon S) = \iota (\epsilon ) + \sigma (S) \in {\mathcal {C}} \subset F$ and for an element $g \in {\mathcal {C}}$ we have $\lambda (g) = g$. Thus the claim follows immediately from the main statement.

*Proof of  ***A2** .  By **A1**, $\psi$ is a well-defined monomorphism and obviously $\psi$ is surjective.

*Proof of  ***A3** .  Since $\iota :F^{\times }\rightarrow C_0$ is a monomorphism, we have, using (2.2), that $F^{\times } = F^{\times }/B^{\times }\cong {\mathcal {C}}_{F^{\times }}(B,F)\subset C_0$ which implies that ${\mathcal {C}}(B,F)={\mathcal {C}}^*(B,F)$.

*Proof of  ***A4** .  Theorem 1.1 implies that ${\mathcal {C}}_B(B,F) \subset {\mathsf {E}} \big ({\mathcal {C}}^* (B,F) \big )$. Conversely, let $\epsilon S \in F$ such that $[\epsilon S] \in {\mathsf {E}} \big ({\mathcal {C}}^* (B,F) \big )$. Then **A2** implies that $\iota (\epsilon ) + \sigma (S) \in {\mathsf {E}} ({\mathcal {C}})$. Thus, by the definition of *B*, it follows that $\epsilon S \in B$ whence $[\epsilon S] \in {\mathcal {C}}_B (B,F)$. $\square$
